# Diagnostic Accuracy of Global Longitudinal Strain for Detecting Exercise Intolerance in Patients with Ischemic Heart Disease

**DOI:** 10.3390/jcdd10010010

**Published:** 2022-12-28

**Authors:** Sisi Zhang, Yujian Liu, Luying Jiang, Zhaozhao Wang, Wanjun Liu, Houjuan Zuo

**Affiliations:** 1 Division of Cardiology, Department of Internal Medicine, Tongji Hospital, Tongji Medical College, Huazhong University of Science and Technology, Wuhan 430030, China; 2 Hubei Key Laboratory of Genetics and Molecular Mechanisms of Cardiologic Disorders, Wuhan 430030, China

**Keywords:** 2D speckle-tracking echocardiography, global longitudinal strain, cardiopulmonary exercise testing, exercise intolerance, ischemic heart disease

## Abstract

Objective: Global longitudinal strain (GLS) is a sensitive and reproducible predictive factor in patients with ischemic heart disease (IHD), although its correlation with exercise tolerance is unknown. We aimed to identify the correlation between global longitudinal strain (GLS) and cardiopulmonary exercise testing (CPX) parameters and assess the prognostic implications and accuracy of GLS in predicting exercise intolerance in populations with ischemic heart disease (IHD) using CPET criteria. Methods: Prospectively, 108 patients with IHD underwent CPX and 2D speckle-tracking echocardiography. Correlation between GLS and multiple CPX variables was assessed using Spearman’s correlation analysis and univariate regression analysis. A receiver operating characteristic (ROC) curve analysis was performed on GLS to detect exercise intolerance. Results: GLS was correlated with peak oxygen uptake (peak VO_2_; r = −0.438, *p* = 0.000), %PPeak VO_2_ (−0.369, *p* = 0.000), peak metabolic equivalents (METs@peak; r = −0.438, *p* < 0.01), and the minute ventilation–carbon dioxide production (VE/VCO_2_) slope (r = 0.257, *p* < 0.01). Weak-to-moderate correlations were also identified for the respiratory exchange rate at the anaerobic threshold (RER@AT), end-tidal carbon dioxide at the anaerobic threshold (PETCO_2_@AT), oxygen consumption at the anaerobic threshold (VO_2_@AT), carbon dioxide production at the anaerobic threshold (VCO_2_@AT), and metabolic equivalents at the anaerobic threshold (METs@AT; *p* < 0.01). On multivariate analysis, the results showed that age, the BMI, and GLS are independent predictors for reduced exercise capacity in patients with IHD (*p* < 0.01). The area under the ROC curve value of GLS for identifying patients with a peak VO_2_ of <14 mL/kg/min was 0.73 (*p* = 0.000). Conclusion: As a sensitive echocardiographic assessment of patients with ischemic heart disease, global longitudinal strain is an independent predictor of reduced exercise capacity and has a sensitivity of 74.2% and a specificity of 66.7% to detect exercise intolerance.

## 1. Introduction

Catheter coronary angiography (CCA), the traditional gold standard for diagnosing coronary artery disease (CAD), identifies visual obstructive lesions [1]. Despite its advantages, the widespread clinical application of CCA remains limited due to the relative risk, technical dependence, and substantial equipment costs. Furthermore, two-thirds of females and one-third of males with stable ischemic heart disease (IHD) have no obstructive CAD on CCA [2], which is associated with worse outcomes. Conventional echocardiography predominantly depends on assessing the left ventricle (LV) ejection fraction (EF) and abnormal wall motion. However, since regional wall motion abnormalities (WMAs) are not evident at rest in approximately 50% of patients, transthoracic echocardiography (TTE) is not diagnostically informative in patients with IHD [3,4,5,6]. Two-dimensional speckle-tracking echocardiographic (2D-STE) imaging is a novel, effective method to measure myocardial deformation and provides a comprehensive quantitative assessment of cardiac function. In patients with ST-elevation myocardial infarction (STEMI), 2D longitudinal strain allows a more objective assessment of myocardial regional and global kinetic injuries and the severity of CAD [7].

Cardiopulmonary exercise testing (CPX) is another non-invasive test that can assess cardiovascular, respiratory, and skeletal physiology. Compared to traditional electrocardiogram (ECG) stress testing, CPX gas exchange variables, such as peak oxygen pulse (O_2_ pulse), peak oxygen uptake (peak VO_2_), peak carbon dioxide exertion (peak VCO_2_), anaerobic threshold (AT), VO_2_/WR, and respiratory equivalent during anaerobic threshold (VE/VCO_2_), provide more sensitive and specific information to detect the onset of ischemia, mortality, and hospitalization [8,9,10,11].

Previous studies have failed to identify the correlation between the EF and peak VO_2_, except for diastolic function and right ventricular (RV) function [12,13,14]. However, these studies predominantly focused on patients with heart failure (HF), and there have been no studies on the correlation between the exercise capacity determined with CPET and 2D-STE in patients with IHD. This study aims to identify and evaluate this correlation to assess whether GLS can predict the exercise capacity and cardiorespiratory fitness (CRF) of these patients.

## 2. Materials and Methods

### 2.1. Study Population

This prospective study was conducted at Tongji Hospital, Tongji Medical College, and Huazhong University of Science and Technology from November 2021 to May 2022.

A total of 108 patients with stable IHD, which also means chronic coronary syndromes (CCS), as defined by the European Society for Cardiology (ESC) guidelines in 2019. The patients were treated through either percutaneous coronary intervention (PCI) or optimal medical therapy following coronary stenosis on cardiac catheterization examination. They also underwent conventional 2D-ECG and symptom-limited CPX within 1 day. Comorbidities and hematological examinations were recorded. The exclusion criteria were patients with acute myocardial infarction or unstable angina in the previous 6 months, reduced LVEF (<40%), intermittent claudication, mitral stenosis, aortic valve disease, atrial fibrillation, and premature ventricular complexes; patients with abnormal resting regional wall motion on ECG; and patients with suboptimal-quality images to assess strain.

### 2.2. Cardiopulmonary Exercise Testing

All patients underwent symptom-limited CPX on a CARDIOVIT CS-200 (Schiller, Barr, Switzerland) on the same day before/after 2D-STE, and the procedure complied with the American Heart Association (AHA) statement concerning exercise Standards for Testing and Training [15,16]. The test was performed using modified Bruce protocols via cycle ergometry with a gradual increase in the work rate within 1 min. The increased value was tailored based on the individual’s physical conditioning and exercise tolerance, resulting in a test duration of 8 to 12 min until the subject could no longer maintain a consistent pedaling frequency [17]. A 12-lead ECG and oxygen saturation were monitored continuously during the test, with blood pressure measured every 2 min. Peak VO_2_ was the average value of the highest 20 s at the last stage of the exercise test and was expressed as absolute (L/min) or relative (mL/kg/min). The VE/VCO_2_ slope was calculated using linear regression during exercise (y = mx + b, m = slope), while the anaerobic threshold (AT) was determined using the V-slope technique [18,19]. The following formula was used to calculate the percentage of predicted peak VO_2_ (%PPeak VO_2_): %PPeak VO_2_ = achieved peak VO_2_/predicted peak VO_2_ obtained using the Wasserman equation × 100 [20]. A %PPeak VO_2_ of <80% is considered the best stratification of patients with functional impairment (New York Heart Association Class II or higher) compared with those without limitations [21]. The slope of the relationship between the rise in VO_2_ over the rate of increase in the work rate (△VO_2_/△work rate) was expressed as follows: △VO_2_/△work rate = (peak VO_2_ − unloaded VO_2_)/[(T − 0.75) × S], where T is the time of incremental exercise and S is the slope of work rate incremental in watts per minute [22].

### 2.3. Conventional Echocardiography

Standardized transesophageal echocardiogram (TEE) examinations were performed using Vivid E9 Ultrasound systems (GE Healthcare Vingmed Ultrasound AS, Horten, Norway) under the guidelines of the American Society of Echocardiography [23]. The electrocardiogram (ECG) data included left end-diastolic dimensions (LVEDD), the LVEF (calculated according to Simpson’s method), peak early diastolic filling (E) and late diastolic filling (A) velocities, the E/A ratio, and early and late diastolic septal mitral annular velocities (E’ and A’, respectively), obtained from the pulsed-wave tissue ratio [24].

### 2.4. Two-Dimensional Speckle-Tracking Echocardiography (2D-STE)

**Figure 1:**
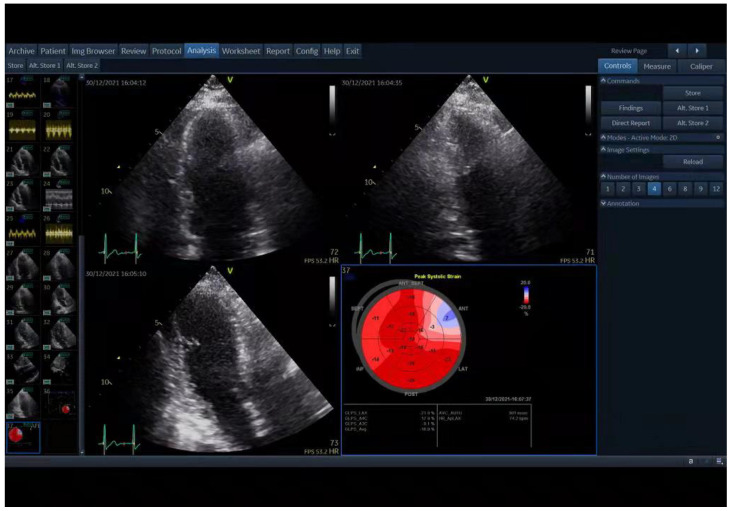
Two-dimensional speckle-tracking echocardiography (2D STE) analysis shows the result of global longitudinal strain (GLS) on a bull’s-eye depiction acquired by EchoPAC.

As previously described, a strain specialist blind to the clinical data of the patients performed longitudinal strain assessments of the LV from three apical views (4-chamber, 2-chamber, and 3-chamber) using EchoPAC (GE Healthcare Vingmed Ultrasound AS) [24]. The endocardial border and myocardium were automatically tracked throughout the cardiac cycle. A region of interest was traced along the endocardial border from an end-systolic frame, and the thickness of the region of interest was adjusted to include the maximum wall thickness. The mean peak longitudinal systolic strain of all LV segments from the three apical views was used to calculate GLS and generated a 17-segment bull’s-eye display (Figure 1). GLS values were presented as negative values. According to a previous report, the normal value of GLS is ≤−17.6 [25].

### 2.5. Statistical Analysis

Continuous data were presented as the mean ± SD or the median (interquartile range, IQR). Categorical variables were expressed as numbers and percentages. Student’s *t*-test and the Mann–Whitney test were performed for quantitative variables, while Pearson’s chi-square test and Fisher’s exact test were performed for categorical variables to compare the differences between the two groups (normal GLS group vs. impaired GLS group). Correlations between GLS and multiple CPX variables were performed using Spearman’s correlation analysis. The significant variables (*p* < 0.05) from the univariate analysis were included in the multiple stepwise regression analysis for assessing independent correlations to peak VO_2_. Receiver operating characteristic (ROC) curves were generated, and area under the curve (AUC) values were calculated to determine the discrimination value of GLS to predict a peak VO_2_ of <14 mL/kg/min. A *p*-value of <0.05 was considered statistically significant. All data were analyzed using SPSS Statistics software. (SPSS 18, SPSS Inc., Chicago, IL, USA).

## 3. Results

### 3.1. Patients’ Baseline Characteristics

Of the 108 patients included in the study, 56.5% (*n* = 61) had impaired GLS (>−17.6), while 43.5% (*n* = 47) had normal GLS (≤−17.6). The baseline characteristics of the two groups are presented in Table 1. No significant differences were found between the two groups except for angiotensin-converting enzyme inhibitor/angiotensin receptor blocker (ACEI/ARB) usage.

### 3.2. ECG and CPX data

ECG and CPX parameters between the two groups are shown in Table 2. Compared with the impaired GLS group, patients with normal GLS exhibited significantly higher VO_2_/kg@AT, Load@AT, VCO_2_/kg@AT, RER@AT, metabolic equivalents (METs)@AT, VO_2_/kg@peak, VCO_2_/kg@peak, RER@peak, and %PPeak VO_2_, which indicated a better exercise capacity. TEE data were similar across both groups.

### 3.3. Correlation of GLS with CPX Variables

**Figure 2:**
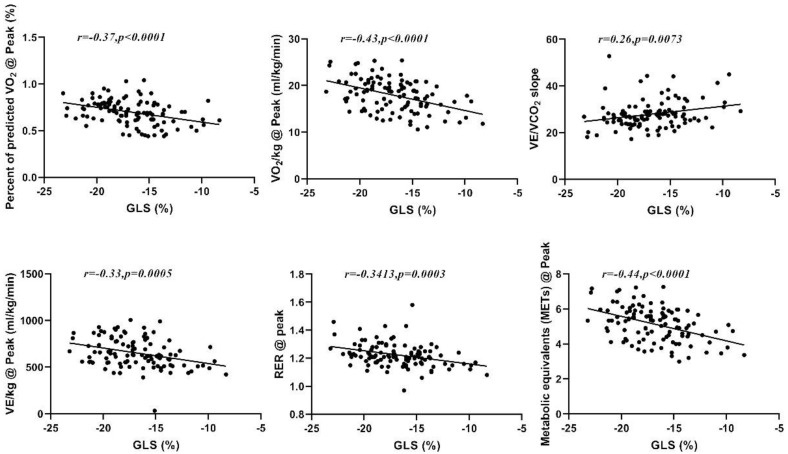
Correlation between GLS and %PPeak VO_2_, peak VO_2_, VE/VCO_2_slope, peak VE, peak RER, and peak METs.

The Pearson correlation of GLS with CPX data (Table 3) revealed that GLS is inversely related to some of the analyzed CPX variables, including RER@peak (r = −0.341, *p* < 001), VO_2_/kg@peak (r = −0.432, *p* < 0.01), METs@peak (r = −0.438, *p* < 0.01), VE/kg@peak (r = −0.328, *p* < 0.01), and %PPeak VO_2_ (r = −0.37, *p* < 0.01), and directly related to VE/VCO_2_slope (r = 0.257, *p* < 0.01); see Figure 2. Correlations were also found for RER@AT, PETCO_2_@AT, VO_2_/kg@AT, VCO_2_/kg@AT, and METs@AT (*p* < 0.01). The EF value showed no significant correlation with any of the analyzed CPET variables (Table 3).

On univariate analysis, the results showed that age (*p* < 0.01) and GLS (*p* < 0.01) appear to be associated with reduced exercise tolerance in subjects with IHD, while on multivariate analysis, age, the BMI, and GLS were independent predictors of reduced exercise capacity (Table 4).

**Figure 3:**
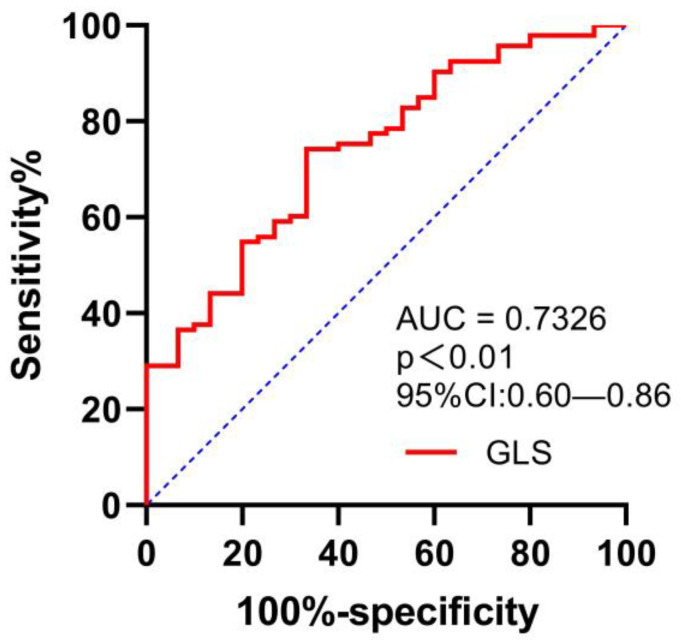
ROC curve for evaluating the ability of GLS in predicting VO_2_ of <14 mL/kg/min.

The area under the ROC curve (AUC) value for GLS in the detection of peak VO_2_ of <14 mL/kg/min was 0.73 (95% confidence interval (CI) 0.6–0.86), with a sensitivity of 74.2% and a specificity of 66.7%, for a cut-off GLS value of −15.2% (*p* = 0.000); see Figure 3.

## 4. Discussion

To the best of our knowledge, this is the first study to describe the relationship between GLS and CPX parameters in the population with IHD. The study highlights weak-to-moderate correlations between GLS and functional CPX parameters and further demonstrates that GLS can detect reduced exercise capacity in these patients.

The non-invasive detection of ischemia for non-obstructive CAD remains a clinical challenge. Previous studies have recognized GLS as one of the most sensitive and reproducible indicators of ischemia and have shown that GLS is superior to the EF in detecting an early reduction in contractile function [26]. The LVEF is not correlated with functional capacity [27]. Most evidence on the association between exercise tolerance and cardiac strain has predominantly focused on patients with HF [28]. However, this relationship has not been investigated and demonstrated in patients with IHD. GLS has emerged as a promising parameter of exercise capacity [29]. This study is the first to show the correlation between GLS and exercise capacity in patients with IHD, which has not been investigated in prior studies of 2D-STE and CPX after a coronary angiogram. More than half of the patients referred for coronary angiography are reported to have normal or non-obstructive CAD, and compared to optimal medical treatment, revascularization is only beneficial in patients with severe ischemia [30,31]. Further coronary angiograms should be considered for symptomatic patients with cardiac dysfunction of reduced peak VO_2_ (<70% of predicted) on CPET. Furthermore, revascularization does not improve the peak VO_2_ for patients with multivessel disease, suggesting that CPET plays a vital role in characterizing the functional consequences of myocardial ischemia [32]. Hence, a more sensitive index for coronary revascularization is needed.

Patients with IHD frequently have reduced exercise capacity, even when the conventional parameters of left ventricular function, such as ejection fraction (LVEF), are within the normal range. In addition, the role of disability, particularly in the context of exercise intolerance, is not fully understood. GLS is associated with the extent of viable myocardial tissue in patients with chronic IHD, where the load has less of an impact. Since sub-endocardial fibers are more sensitive to ischemia, numerous studies have demonstrated that GLS is more accurate at detecting early myocardial disturbances caused by ischemia compared with the EF value [3,25]. Previous research has suggested that GLS is related to the ability to exercise through poor contractile reservation during exercise [33]. Peak VO_2_ and VE/VCO_2_ slope are critical parameters in the detection of obstructive CAD, and in this study, patients with normal GLS had a higher peak VO_2_ (19.19 ± 3.42 mL/kg/min vs. 17.01 ± 3.22 mL/kg/min), and compared with the resting EF, GLS showed a relationship with peak VO_2_ (r = −0.432, *p* < 0.01) and the VE/VCO_2_ slope (r = 0.257, *p* = 0.000). Moreover, multivariable analysis demonstrated that GLS is independently associated with reduced peak VO_2_. Thus, reduced GLS may be an effective indicator of exercise intolerance in this group of patients.

Peak VO_2_, defined as cardiorespiratory fitness (CRF), is a vital clinical sign of all-cause and cardiovascular mortality in patients with cardiovascular diseases, as well as in healthy individuals [34]. Reduced peak VO_2_ is recognized as an independent risk factor for adverse cardiovascular events in populations with IHD. The correlations between GLS and exercise capacity identified in this study further highlight the potential importance of early detection of LV dysfunction in individuals with IHD with exercise intolerance.

Ng et al. reported that GLS at rest was −16.3 ± 2.4 in patients with CAD vs. −19.1 ± 2.9 in patients with non-significant CAD [35]. Similar results were obtained by Biering-Sørensen et al. [36], Gaibazzi et al. [37], Evensen et al. [38], and Shimoni et al. [39]. In this study, the cut-off value of GLS to detect a peak VO_2_ of <14 mL/min/kg was −15.2, with a sensitivity of 74.2% and a specificity of 66.7%. Collectively, these findings indicate the quantifiable and prognostic significance of GLS as a suitable alternative to evaluate patients with reduced exercise capacity.

The value of using CPX in detecting macrovascular ischemia has been previously reported [9]. However, the direct measurement of CPX requires specialized equipment and trained personnel to accurately interpret the results. In addition, patients may be unable or unwilling to undergo this testing. Thus, CPX remains underused in China. Simultaneously, despite numerous attempts to develop surrogates and regression models based on non-experimental test data to predict peak VO_2_, the models are not specific enough to classify CRF as routine practice. Hence, determination of the relationship between GLS and exercise intolerance could potentially allow the prediction of CRF of patients with IHD based solely on ECG-derived GLS. The exercise intolerance prediction results from this study extend the findings of Maia et al. [40] regarding GLS measured in patients with systolic heart failure. In this study, the GLS cut-off value for detecting a peak VO_2_ of <14 mL/min/kg was −15.2. Therefore, GLS could be a valuable tool to discriminate patients with normal exercise capacity from those with reduced exercise capacity.

### 4.1. Limitations

Despite the valuable findings of this study, there are several significant limitations that should also be considered. First, as a single-center study, the small size of the population in the study may limit the generalizability of the findings. Second, more than 50% of the participants in both experimental groups were taking β-receptor blocker medication, a primary treatment for IHD, which significantly lowers the peak VO_2_ in CPX. Further studies should be conducted on subjects using 3D STE.

### 4.2. Conclusions

The study presented the value of GLS measured with 2D speckle-tracking echocardiograph in patients with IHD. The assessment of GLS was able to detect exercise intolerance and identify what has a poor prognosis.

## Figures and Tables

**Table 1:** Baseline characteristics stratified by a GLS median of −17.6%.

Variable	GLS ≥ −17.6 (*n* = 61)	GLS < −17.6 (*n* = 47)	*p*-Value
Age (years)	57.21 ± 10.89	60.96 ± 5.95	0.025
Gender (%)			
Male	39 (63.93)	28 (59.57)	0.643
Female	22 (36.07)	19 (40.43)	0.643
Height (cm)	164.74 ± 7.19	163.12 ± 7.64	0.262
Weight (kg)	163.12 ± 7.64	66.29 ± 11.20	0.1
BMI (kg/m^2^)	25.65 ± 2.97	24.80 ± 3.03	0.145
Comorbidities (%)			
Hypertension	31 (50.82)	30 (63.83)	0.176
Diabetes	18 (29.51)	13 (27.66)	0.833
Dyslipidemia	24 (39.34)	13 (27.66)	0.205
SBP (mmHg)	135.79 ± 28.73	142.04 ± 15.52	0.151
DBP (mmHg)	73.74 ± 15.21	73.55 ± 9.36	0.942
HR (bpm)	101.33 ± 13.30	98.28 ± 12.24	0.224
Medications (%)			
β-Blocker	38 (62.30)	24 (51.06)	0.242
ACE inhibitors/ARB	22 (36.07)	26 (55.32)	0.046 *
Statin	57 (93.44)	47 (100.00)	0.074
Aspirin	57 (93.44)	44 (93.62)	0.971
Clopidogrel	55 (90.16)	46 (97.87)	0.107
CCB	14 (22.95)	14 (29.79)	0.422
Serum marker			
Creatinine (umol/L)	82.03 ± 23.00	76.82 ± 20.28	0.233
CK (mmol/L)	258.11 ± 802.31	96.77 ± 42.57	0.15
Cholesterol (mmol/L)	4.39 ± 1.30	4.05 ± 1.12	0.155
Glucose (mmol/L)	6.320 (5.4, 8.4)	6.510 (5.9, 8.2)	0.479
HDL (mmol/L)	1.050 (0.9, 1.3)	1.080 (0.9, 1.2)	0.942
LDL (mmol/L)	2.67 ± 1.03	2.35 ± 0.94	0.111
Lipoprotein (a) (mmol/L)	40.12 ± 47.97	45.81 ± 57.46	0.59
Triglycerides (mmol/L)	1.560 (1.0, 2.7)	1.600 (0.9, 2.7)	0.829
Hemoglobin (g/L)	137.49 ± 16.41	135.30 ± 16.79	0.507
NT-proBNP	315.26 ± 789.62	286.42 ± 1077.99	0.898
IHD (%)			
Non-PCI	27 (44.26)	28 (59.57)	0.115
PCI	34 (55.74)	19 (40.43)	0.115

Abbreviations: GLS, global longitudinal strain; BMI, body mass index; SBP, systolic blood pressure; DBP, diastolic blood pressure; HR, heart rate; ACE, angiotensin-converting enzyme; ARB, angiotensin II receptor blockers; CCB, calcium channel blocker; CK, creatine kinase; HDL, high-density lipoprotein; LDL, low-density lipoprotein; NT-proBNP, N-terminal pro-B-type natriuretic peptide; IHD, ischemic heart disease; PCI, percutaneous intervention. * *p* < 0.05.

**Table 2:** Echocardiographic characteristics, global longitudinal strain, and primary CPET variables between groups.

Variables	GLS ≥ −17.6 (*n* = 61)	GLS < −17.6 (*n* = 47)	*p*-Value
GLS (%)	−14.68 ± 2.16	−19.61 ± 1.45	0.000
LVEDD (mm)	47.68 ± 4.84	47.14 ± 4.68	0.568
LVEF (%)	0.580 (0.5,0.6)	0.630 (0.6, 0.7)	0.003
E (cm/s)	71.53 ± 24.19	71.02 ± 19.26	0.911
A (cm/s)	83.96 ± 24.05	75.27 ± 20.77	0.061
E/A	0.94 ± 0.44	0.96 ± 0.28	0.796
E’ (cm/s)	5.84 ± 1.76	6.23 ± 1.58	0.254
A’ (cm/s)	9.58 ± 2.28	9.64 ± 2.43	0.909
RER@ AT	1.02 ± 0.07	1.05 ± 0.06	0.030 *
VO_2_/kg@ AT (mL/kg/min)	11.32 ± 1.92	12.22 ± 1.59	0.010 *
Load@ AT (w)	52.52 ± 19.90	52.91 ± 13.11	0.908
VE@ AT (L/min)	24.80 ± 5.04	24.27 ± 4.42	0.573
VE/kg@ AT (mL/kg/min)	351.52 ± 73.47	368.50 ± 55.74	0.19
VCO_2_/kg@ AT (mL/kg/min)	11.57 ± 2.13	12.83 ± 2.08	0.003 **
HR@ AT (beats)	101.33 ± 13.30	98.28 ± 12.24	0.224
Metabolic equivalents@ AT (Mets)	3.24 ± 0.55	3.50 ± 0.45	0.009 **
RER@ peak	1.21 ± 0.09	1.24 ± 0.08	0.028 *
VO_2_/kg@ peak (mL/kg/min)	17.01 ± 3.22	19.19 ± 3.42	0.001 **
VO_2_ peak/predicted	0.66 ± 0.14	0.76 ± 0.09	0.000 **
Load@ peak (w)	88.39 ± 30.73	92.60 ± 24.83	0.446
VE@ peak (L/min)	43.79 ± 10.89	45.82 ± 11.88	0.358
VE/kg@ peak (mL/kg/min)	622.35 ± 159.42	691.72 ± 145.81	0.022 *
VCO_2_/kg@ peak (mL/kg/min)	20.65 ± 4.55	24.01 ± 5.30	0.001 **
HR@ peak (beats)	123.97 ± 18.77	126.09 ± 21.18	0.584
Metabolic equivalents@ peak (Mets)	4.86 ± 0.92	5.49 ± 0.97	0.001
VE/VCO_2_ slope	28.91 ± 5.83	26.65 ± 5.98	0.051
dVO_2_/d Work rate (mL/min/watt)	9.19 ± 1.77	9.72 ± 1.64	0.112
FEV1 (L)	2.44 ± 0.70	2.47 ± 0.61	0.8
FVC (L)	3.05 ± 0.79	3.13 ± 0.80	0.622
FEV1/FVC (%)	0.80 ± 0.09	0.80 ± 0.10	0.978
VC max	3.19 ± 0.79	3.23 ± 0.78	0.784

Abbreviations: LVEDD, left ventricular end-diastolic diameter; LVEF, left ventricular ejection fraction; RER, respiratory exchange ratio; AT, anaerobic threshold; VO_2_, oxygen uptake; VCO_2_, ventilatory carbon dioxide; HR, heart rate; VE, exercise ventilation; FEV1, forced expiratory volume in 1 s; FVC, forced vital capacity; VC, vital capacity. * *p* < 0.05, ** *p* < 0.01.

**Table 3:** Correlations between numerical parameters of CPX with the left ventricular ejection fraction (LVEF) and global longitudinal strain (GLS).

Values	GLS		EF	
	r	*p*-Value	r	*p*-Value
dVO_2_/dWR (mL/min/W)	−0.177	0.067	−0.023	0.82
VE @AT (L/min)	0.007	0.944	−0.064	0.526
HR @AT (bpm)	0.151	0.118	−0.007	0.445
Load @AT (watts)	−0.04	0.682	0.037	0.711
RER@ AT	−0.305	0.001 **	0.081	0.421
PETCO_2_ @AT (mmHg)	−0.274	0.004 **	0.187	0.062
PETO_2_ @AT (mmHg)	0.047	0.63	−0.086	0.394
Systolic BP@A (mmHg)	−0.077	0.431	0.144	0.151
Diastolic BP@AT (mmHg)	0.019	0.848	0.028	0.784
VE/kg @AT (mL/kg/min)	−0.158	0.102	0.097	0.335
VO_2_/kg @AT (mL/kg/min)	−0.267	0.005 **	0.202	0.042
VCO_2_/kg @AT (mL/kg/min)	−0.335	0.000 **	0.192	0.054
Metabolic equivalents (METs)@ AT	−0.271	0.005 **	0.205	0.04
VE @peak (L/min)	−0.2	0.038	−0.077	0.445
HR @peak (bpm)	−0.098	0.313	−0.087	0.39
Load @peak (watts)	−0.151	0.118	−0.025	0.808
RER@ peak	−0.341	0.000 **	0.016	0.875
PETCO_2_ @peak (mmHg)	−0.244	0.011	0.078	0.438
PETO_2_ @peak (mmHg)	0.035	0.717	−0.126	0.209
Systolic BP@peak (mmHg)	−0.167	0.085	0.131	0.19
Diastolic BP@peak (mmHg)	−0.093	0.339	−0.031	0.762
VE/kg @peak (mL/kg/min)	−0.328	0.001 **	0.035	0.725
VO_2_/kg @peak (mL/kg/min)	−0.432	0.000 **	0.075	0.459
VCO_2_/kg @peak (mL/kg/min)	−0.456	0.000 **	0.068	0.499
Metabolic equivalents (METs)@ peak	−0.438	0.000 **	0.076	0.448
VE/VCO_2_slope	0.257	0.007 **	−0.242	0.015
%PPeak VO_2_ (%)	−0.369	0.000 **	0.135	0.178
VC max (L)	−0.087	0.369	−0.045	0.655
FEV1 (L)	−0.024	0.808	−0.009	0.931
FVC (L)	−0.1	0.308	0.004	0.966
FEV1/FVC (%)	0.116	0.238	−0.019	0.852

Abbreviations: dVO_2_/dWR: oxygen-consumption-to-work-rate ratio; VE, exercise ventilation; HR, heart rate; RER, respiratory exchange ratio; AT, anaerobic threshold; PETCO_2_, end-tidal carbon dioxide; PETO_2_, end-tidal partial pressures of oxygen; BP, blood pressure; VO_2_, oxygen uptake; VCO_2_, ventilatory carbon dioxide; FEV1, forced expiratory volume in 1 s; FVC, forced vital capacity; VC, vital capacity. ** *p* < 0.01.

**Table 4:** Univariate and multivariate predictors to predict reduced exercise capacity (peak VO_2_) in patients with IHD.

Variables	Univariate Analysis			Multivariate Analysis		
	OR	95%CI	*p*-Value	OR	95%CI	*p*-Value
Gender	4	1.258–12.72	0.019	3.998	0.766–20.859	0.1
Age	0.869	0.791–0.954	<0.01	0.793	0.684–0.919	<0.01
BMI	0.824	0.681–0.997	0.047	0.663	0.487–0.903	<0.01
EF	0.975	0.010–97.809	0.991	0.028	0.000–4.464	0.167
GLS	0.737	0.606–0.896	<0.01	0.618	0.445–0.859	<0.01
LV	0.946	0.844–1.060	0.34	0.876	0.731–1.05	0.152

Abbreviations: BMI, body mass index; EF, ejection fraction; GLS, global longitudinal strain; LV, left ventricular; OR, odds ratio; CI, confidence interval.

## Data Availability

Data is unavailable due to privacy or ethical restrictions.
